# Predict Disease Progression With Reaction Rate Equation Modeling of Multimodal MRI and PET

**DOI:** 10.3389/fnagi.2018.00306

**Published:** 2018-10-08

**Authors:** Li Su, Yujing Huang, Yi Wang, James Rowe, John O’Brien

**Affiliations:** ^1^Department of Psychiatry, University of Cambridge, Cambridge, United Kingdom; ^2^China-UK Centre for Cognition and Ageing Research, Faculty of Psychology, Southwest University, Chongqing, China; ^3^Department of Clinical Neurosciences, University of Cambridge, Cambridge, United Kingdom

**Keywords:** MRI, PET, computational modeling, disease progression, AD, dementia

## Abstract

Neurodegenerative dementia often has multiple types of underlying pathology, for example, beta-amyloid, misfolded tau, chronic neuroinflammation and neurodegeneration may coexist in Alzheimer’s disease. However, the relationship between them is often unclear, in other words, whether one pathology is upstream or downstream of others can be very difficult to investigate directly. This is partly because the underlying pathology in dementia may precede detectable symptoms by several years if not decades. The time scale associated with disease progression in dementia generally exceeds that in conventional longitudinal imaging studies in humans, so it is difficult to directly observe the temporal ordering of different pathologies. Also, animal studies are not always transferable to patients due to obvious differences between the two systems. To investigate the disease progression and relationships among underlying pathological changes, we propose a novel computational modeling approach for multimodal MRI and PET inspired by *reaction rate equation* in chemical kinetics. We also discuss the possibility and prerequisites to use cross-sectional data to generate preliminary hypothesis for future longitudinal studies. It has been shown that the rate of change in some biomarkers can be approximated by the average trajectory across patients at different stages of disease severity in cross-sectional studies. The relationship modeled in our approach is akin to that in the control theory, and can be assessed by demonstrating that the presence of one disease related biomarker predicts dynamics in another. We argue that the proposed framework has important implications for trials targeting different pathologies in dementia.

## Introduction

Previous studies have shown that beta-amyloid, tau, neuroinflammation and neurodegeneration all play a significant role in the etiology of Alzheimer’s disease (AD), but little is known about their relationships (Edison et al., 2008; Lehmann et al., 2013). In particular, whether one type of pathology is the upstream or downstream event to others has significant impact on future trials appropriately targeting them at the right point in the disease course (Jack et al., 2013). In addition, systematically determining potential treatment targets in diseases with multiple interacting pathologies has strategic importance for effective treatments. In the case of AD, beta amyloid has been regarded as an early event in the disease progression therefore making it one of the potential targets (Jack et al., 2013). However, the failures of several recent trials of anti-amyloid therapy (Le Couteur et al., 2016) may arguably be caused by the drugs were given too late in the disease course to be effective. The co-existence of other interacting pathologies might also reduce the efficacy of those anti-amyloid drugs. So, investigating the relationship between multiple pathologies and their associated imaging biomarkers in dementia and determining alternative or complementary treatment targets are necessary.

The relationship among more than one type of pathology has primarily studied in animal models of AD, however, due to the differences in techniques, the findings from those animal research were largely inconsistent (Yoshiyama et al., 2007). In one study, activated microglia was found to facilitate the propagation of misfolded tau in mouse brains (Wes et al., 2014), and after depletion of microglia in the mouse brain, the spread of tau from the entorhinal cortex to the dentate gyrus was significantly decrease (Asai et al., 2015). This evidence points to a causal effect of neuroinflammation on the phosphorylation or propagation of tau as microglia activations seems both sufficient and necessary for tau phosphorylation and its *trans-*synaptic propagation. Although some studies showing microglia activation preceded tangle formation in P301S transgenic mice with overexpressed mutant human tau, opposite pattern was found in Cx3cr1 mice with tau deficiency that shows tau phosphorylation without significant microglia activation and reduced neuroinflammation (Yoshiyama et al., 2007). In addition, the obvious differences between humans and animals limit the ability to translate findings from the modal systems to human patients. To see how different types of pathology interact in humans, it has required a multimodal imaging study with PET and MRI in the same cohort of participants to reveal the potential influences among them. Recently such data is emerging, but the analysis and the inference frameworks still lag behind in characterizing multimodal and longitudinal imaging data in patients with dementia.

In neurodegenerative dementia such as AD, the development of underlying pathology takes several years if not decades before any detectable symptoms occur (Jack et al., 2013), so it is challenging to study in humans using conventional longitudinal design. This is because it is costly and difficult to follow large cohort of healthy participants free from AD pathologies over many decades with only very small proportion of them eventually develops dementia. For imaging studies, the MR scanner will also unavoidably change over time, making the data less comparable at different time points. As a result, existing longitudinal human imaging data only tracks a relatively short period (e.g., several years) within the evolution of the disease in patients with dementia (Ishiki et al., 2015). In addition, different biomarkers may have different sensitivity to the underlying pathology. So comparing biomarkers obtained from multimodal imaging is nontrivial. With the absence of suitable longitudinal data tracking the long-term evolution of dementia in humans and the inconsistent evidence from animal models, studying interaction among factors of dementia may sometimes rely on cross-sectional data and by modeling the relationship within clinical populations representing different severities and stages of disease progression. Thus, ideal analysis methods for longitudinal data must also consider cross-sectional data to be widely applicable in dementia research.

Here, we proposed a novel computational modeling approach based on *reaction rate equation* modeling in chemical kinetics to infer relationship between more than one types of imaging biomarker in dementia. The specific type of relationship in this model was defined in a control theory sense (Friston et al., 2016) meaning whether the presence of one disease related pathological process (e.g., tau) in the past predicts to the dynamics in another (e.g., beta-amyloid, microglia activation, and neurodegeneration) expressed using differential equations (Yang et al., 2011; Villemagne et al., 2013; Young et al., 2014; Budgeon et al., 2017; Lturria-Medina et al., 2017; Oxtoby et al., 2018).

## The Longitudinal Model

With the advances of imaging technology, many types of pathology can be measured *in vivo* using multimodal MRI and multi-tracer PET within the same cohort (Passamonti et al., 2016, 2018; Su et al., 2016). For example PET data is often in the form of binding potential or SUVR that are proxies for the concentration of a substance, e.g., some neurotoxic proteins. MRI data is often in the form of gray matter volume as well as cortical thickness. For simplicity, we will illustrate the approach by the interaction between two substances measured from PET imaging, each associated with a specific pathology. However, this model can easily be extended to MRI and between PET and MRI.

Specifically, if one substance *BP*_1_ (related to one pathology) is the upstream species of another substance *BP*_2_ (related to a different pathology) in a biochemical reaction, we can express this as Equation 1 (which is called balance equation).

(1) $${\mathrm{aBP}}_{1}+b_{\text{i}}\Sigma B_{\text{i}}\to {\mathrm{mBP}}_{2}+n_{\text{j}}\Sigma C_{\text{j}}$$

Where *B*_i_ and *C*_j_ represent a set of unknown substances involved in this process and *a, b*_i_, *m* and *n*_j_ represent a set of unknown coefficients. Also, *i* and *j* are indices of these unknown substances of which the number of them are also unknown. Here, we do not assume this process is a single step reaction nor substance represented by *BP*_1_ directly turns into *BP*_2_. It can be seen that Equation 1 describes a specific form of relationship, i.e., *BP*_1_ is an up-stream event of *BP*_2_ instead of the vice versa. This model is difficult to estimate because it contains too many free parameters that we cannot evaluate empirically in patients. It will be discussed later that Equation 1 remains useful to conceptualize the relationship between multiple types of pathology in the disease.

To reduce the number of free parameters in the model, we will use chemical kinetics to calculate the speed of the reaction. Here, we do not need to estimate the concentration of all products in *C*_j_ if we can instead measure the speed at which *BP*_2_ is produced because it will be perfectly correlated with the speed at which the reaction happens. In chemical kinetics, the speed at which a reaction happens is expressed using the reaction rate equation (Equation 2).

(2) $$\frac{d[{\mathrm{BP}}_{2}]}{\mathrm{dt}}=-\frac{d[{\mathrm{BP}}_{1}]}{\mathrm{dt}}=k{\left[{\mathrm{BP}}_{\text{l}}\right]}^{\text{x}}\prod {\left[B_{\text{i}}\right]}^{\text{y}}$$

Where *k* is a reaction rate constant, x and y are unknown reaction orders, and the concentrations [*BP*_1_] and [*BP*_2_] can be measured by PET from human participants, example of which are [^18^F]AV1451 for tau, [^11^C]PiB for beta-amyloid and [^11^C]PK11195 for activated microglia. [*A*] denotes the concentration of substance *A* while [*A*]_0_ represents the initial value of the concentration at a predefined baseline time point. In most diseases, the initial concentration of disease related substance [*BP*_1_]_0_ is significantly lower compared with other substances [*B_i_*]_0_ that were already in the brains of healthy subjects (i.e., [*B_i_*]_0_ > > [*BP*_1_]_0_), we can apply the pseudo 1st order approximation in chemical kinetics and simplify Equation 2 to Equation 3 that only contains the concentration [*BP*_1_]. It should be noted that the validity of this assumption is also depending on the selection of the initial state. For AD, the MCI stage is a relatively reasonable and clinical defined initial state for the disease process, however, as new method for the early detection of AD emerges, a more accurate initial state could be defined in the future. Thus, for the longitudinal model, an ideal baseline state is at the very early point of the diseases. As previously mentioned, longitudinal data with such suitable baseline is still lacking.

(3) $$\frac{d[{\mathrm{BP}}_{2}]}{\mathrm{dt}}=k{\left[{\mathrm{BP}}_{1}\right]}^{x}\prod {\left[B_{\text{i}}\right]}_{0}^{\text{y}}=k^{\prime }{\left[{\mathrm{BP}}_{1}\right]}^{\text{x}}$$

Where *k*′ is a constant because we assume that the initial concentrations of [*B_i_*]_0_ are also constants. Finally, we apply natural logarithm on both sides of the non-linear Equation 3, which is more difficult to model and evaluate, resulting an asymmetric linear model (Equation 4), which can be statistically tested using linear regression as we explained in subsequent sections.

(4) $$\ln(\frac{d[{\mathrm{BP}}_{2}]}{\mathrm{dt}})=\ln(k^{\prime }{\left[{\mathrm{BP}}_{1}\right]}^{\text{x}})=\ln(k^{\prime })+x\ln([{\mathrm{BP}}_{1}])$$

## The Cross-Sectional Model

As previously discussed, the progression of dementia often takes years or decades, thus conventional longitudinal data are either unavailable or unable to capture the full dynamic changes and temporal ordering in underlying pathologies. Thus, short-term follow-up data was often used to fit the common biomarker trajectory building up a complete picture of the population time course over a longer term (Budgeon et al., 2017). Although the longitudinal model is still more powerful and likely to be more accurate in revealing relationship between biomarkers, the method proposed here should be considered with respect to cross-sectional data when longitudinal data is not available see similar approached used by Young et al. (2014). This allows us to generate hypothesis from existing cross-sectional studies, and then to test it with longitudinal data in the future.

In some cases, the average rate of change in tau pathology over time has been shown to be consistent with their rate of change across individual subjects with different disease severity or disease progression score (Ishiki et al., 2015). In the cross-sectional model under the above condition, we replace the total derivative with respect to time in Equation 4 by partial derivative with respect to an appropriate measurement of disease severity or cognitive functioning (denoted by τ); see Equation 5.

(5) $$\ln(\frac{\partial [{\mathrm{BP}}_{2}]}{\partial \tau })=\ln(k^{\prime })+x\ln([{\mathrm{BP}}_{1}])$$

In this model, we approximate the rates of longitudinal change in regional PET binding and GM density for a cohort of patients by the slope parameters with respect for the disease severity score or cognition derived from a multiple linear regression model. Here, we assumed that the pathological changed linearly with the disease severity of cognitive measure. This assumption although not true in the absolute sense, it avoids over-fitting the noise when the sample size is limited, for example in most PET studies. In order to control other known confounding factors, one can include subject’s age, sex, and years of education as covariates in the regression analysis.

## Statistical Analysis

A critical step for computational modeling is empirical validation, in other words, whether the proposed model explains human data. So, one should fit the reaction rate model to the imaging data, either longitudinal or cross-sectional. As the model is a linear equation, the “goodness of fit” can be statistically evaluated by linear correlation. In other words, if we hypothesize that one type of pathology is the upstream event of another under the assumption and formulation of this model, baseline level of the former should be correlated with the rate of changes in the latter either over time for longitudinal data or across different degrees of dementia severities for cross-sectional data. This approach can be extended in several ways. In addition, we can also apply the modeling to the clinical and cognitive data. Although they cannot be directly formulated within the context of chemical reactions, the quantitative method does allow inference to be drawn between these metrics and brain imaging or other biomarkers.

The statistical tests for longitudinal and cross-sectional data may be different. In the longitudinal case, the dynamic model can be fitted to each individual subject’s time course data, hence the group statistics can be computed using a random effect model across multiple subjects after the model fitting at the individual level for each brain area. However, in the case of cross-sectional data or longitudinal data with short-term follow-up, the rate of biomarker changes can only be estimated for the cohort as a whole. Thus, the test for the fitness of the model cannot be done using the methods for longitudinal data. Instead, it can be done across different brain regions using repeated measure statistics, and the inference can only be drawn for the whole brain. In addition, for the cross-sectional model, including cognitive or clinical data is more difficult than the longitudinal design because the rates of the biomarker changes are estimated for the group rather than for the individuals.

Another challenging issue in computational modeling is model comparison. Different methods have been proposed to account for this issue, such as Bayesian model selection, which gained increasing popularity in imaging analysis and generative modeling in computational neuroscience (Wasserman, 2000). These sophisticated methods deal with difference in model complexity, i.e., the sampler model is the superior model compared with a more complex model if both models can explain the data equally well. This can be understood by the intuitive Occam’s Razor principle, i.e., preferring the most parsimonious model explaining the same variance. However, in our formalism, all models have not only the same number of parameters but also the same analytical form (Equation 4 or 5). As the complexities between alternative models are identical, model comparison is trivial and can be done by simply comparing the goodness of fit.

## Discussion

It is widely accepted that longitudinal imaging is very important for the understanding of disease progression, staging pathology, differential diagnosis, and determining prognosis during clinical trials. Multimodal imaging including structural MRI, DTI, functional MRI and multiple tracers PET has also gained the attention because it allows complex etiologies behind dementing diseases to be studied (Mak et al., 2014). However, the mainstream analysis methods are still limited in their capacity to handle longitudinal data and to systematically relate or combine data from multiple imaging modalities. Moreover, mechanistic interference on the interactions among different pathologies and their associated biomarkers cannot be reliably drawn for the following reasons. First, the majority of imaging data on dementia is either cross-sectional or only tracks the disease progression for a few years and often during the relatively late stages, e.g., after significant cognitive impairments and brain damages have occurred. Such short-term follow-up data may be difficult to reliably capture critical events during the disease course, in particular during the pre-symptomatic stages. Second, existing imaging data is often acquired from single site with relatively small heterogeneous samples. The individual differences among patients are often dramatic because the variation in clinical diagnosis criteria and age as well as the possibility of mixed pathologies or even misdiagnosis between dementia sub-types.

To analyze and model longitudinal and multimodal imaging data with these limitations, several methods have been proposed. For example, imaging biomarkers of pathology measured at different time points from different individuals can be mapped onto a hypothetical time axis representing ‘time-to-dementia’ (Bateman et al., 2012). This approach allows us to normalize different biomarkers from the heterogeneous sample to a single continuous dimension so that temporal ordering can be inferred. Other approaches use data-driven methods to model biomarkers from different data points as discrete events in the disease course. The temporal ordering of events can then be inferred based on the co-occurrence between each pair of different events and Markov chain Monte Carlo methods (Young et al., 2014; Oxtoby et al., 2018).

One advantage of our approach is that the hypothesis about the relationship between different imaging biomarkers are expressed explicitly as chemical reactions, i.e., using the balance equation (Equation 1). Hence, underlying assumptions have to be made explicit, an advantage of most computational models. In this formalism, it is apparent that the temporal ordering between different biomarkers cannot be inferred from the quantity difference between each modeled substance alone. For example, a greater concentration or binding potential in *BP*_1_ than in *BP*_2_ does not always imply that pathology related to *BP*_1_ is the upstream event of *BP*_2_. This is because that the coefficients such as *a* and *m* in Equation 1 are unknown, so it is possible that a small quantity of one substance at upstream results in a larger quantity of another downstream, i.e., when *a > m* in Equation 1. This may give the false inference that the downstream event related to *BP*_2_ is preceding the upstream event related to *BP*_1_. By the same token, the temporal ordering of the biomarkers cannot be solely determined from the spatial extent which is another common way to measure quantity in neuroimaging.

Finally, we argue that this dynamic perspective in modeling biomarkers may be extended in order to be applied to not only neurodegenerative diseases but also neurodevelopmental conditions such ADHD and autism spectrum disorders as well as normal development and aging. However, the limitation of our methods is that it still assumes stationarity, i.e., the rate of change in biomarkers (i.e., the 2nd order derivatives) does not change over time. Future developments are needed to capture the nonlinearity of the disease progression and heterogeneity of the samples. Last but not least, this highly novel approach requires extensive empirical validations using suitable longitudinal and cross-sectional data from different diseases and ideally from cohorts of different age ranges.

## Author Contributions

LS designed the study, developed the computational model, and wrote the manuscript. YH and YW co-developed the computational model. JR and JO’B co-designed the study, obtained funding for the project, and assisted with writing of the manuscript.

## Conflict of Interest Statement

JO’B has been a consultant for GE Healthcare, TauRx, Axon, Axovant, and Lilly and received honoraria for talks from Piramal. The remaining authors declare that the research was conducted in the absence of any commercial or financial relationships that could be construed as a potential conflict of interest.
